# Optical Setup for Error Compensation in a Laser Triangulation System

**DOI:** 10.3390/s20174949

**Published:** 2020-09-01

**Authors:** Patrick Kienle, Lorena Batarilo, Markus Akgül, Michael H. Köhler, Kun Wang, Martin Jakobi, Alexander W. Koch

**Affiliations:** Institute for Measurement Systems and Sensor Technology, Department of Electrical and Computer Engineering, Technical University of Munich, 80333 Munich, Germany; lorena.batarilo@tum.de (L.B.); markus.akguel@tum.de (M.A.); michael.koehler@tum.de (M.H.K.); kun88.wang@tum.de (K.W.); m.jakobi@tum.de (M.J.); a.w.koch@tum.de (A.W.K.)

**Keywords:** laser triangulation, absolute distance measurement (ADM), laser stability, measurement error, temperature stabilization, laser drift, error compensation, drift compensation

## Abstract

Absolute distance measurement is a field of research with a large variety of applications. Laser triangulation is a well-tested and developed technique using geometric relations to calculate the absolute distance to an object. The advantages of laser triangulation include its simple and cost-effective setup with yet a high achievable accuracy and resolution in short distances. A main problem of the technology is that even small changes of the optomechanical setup, e.g., due to thermal expansion, lead to significant measurement errors. Therefore, in this work, we introduce an optical setup containing only a beam splitter and a mirror, which splits the laser into a measurement beam and a reference beam. The reference beam can then be used to compensate for different error sources, such as laser beam dithering or shifts of the measurement setup due to the thermal expansion of the components. The effectiveness of this setup is proven by extensive simulations and measurements. The compensation setup improves the deviation in static measurements by up to 75%, whereas the measurement uncertainty at a distance of 1 m can be reduced to 85 μm. Consequently, this compensation setup can improve the accuracy of classical laser triangulation devices and make them more robust against changes in environmental conditions.

## 1. Introduction

Absolute distance measurement (ADM) has been an essential research field in the past and present [1,2]. Well-known applications using ADM include monitoring of huge constructions [3,4], mechanical design, quality inspection, and manufacturing automation [5,6]. The absolute distance to an object can be measured with several optical techniques including interferometric methods, the time-of-flight principle, phase-detection methods, or laser triangulation [7]. The proper choice of measurement principle depends on several factors like required accuracy, range or measurement rate, and the given environmental conditions. In a laser triangulation device (LTD) simple geometric relations are used to calculate the absolute distance [8,9]. It is a commonly used method in industrial applications due to its simple, cheap, and robust operation and high measurement rates [10]. Disadvantages include the decreasing resolution with increasing measurement distance and its strong dependency on the pointing stability of the laser source as well as the surface properties of the measurement object [11,12]. Most commercialized triangulation systems typically have a relative precision of around $10^{-3}$ to $10^{-4}$ at a working distance of 20–1000 mm with a measurement range of ±1 to ±250 mm [13], though there are a few systems available with a measurement range of up to several meters [2]. Liebe et al. [14] recently introduced a laser triangulation system for a measurement range of 0.5–10 m, achieving an accuracy of around 1.5 mm at a distance of 1 m.

Parameters limiting the accuracy of an LTD can be divided into the laser source [15], air refractive index [5], measurement surface [16,17], optomechanical setup [18], and centroid calculation [19]. Several techniques were introduced in the past to try to eliminate those limitations or to improve the overall setup. The following literature is not exclusively aimed at the improvement of LTDs or distance measurement in general, so some of it is regarding the improvement in different measurement fields, e.g., straightness measurement. Yet those ideas could be used for an LTD.

The influence of the laser beam pointing stability can be eliminated by splitting up the laser beam and monitoring one beam with a reference detector [20], control the beam with an active laser beam stabilization system [21,22] or a thermo-mechanical beam drift stabilization [23], as well as splitting up the laser beam into two parallel beams [15,24,25]. The latter of those options also compensates for errors due to atmospheric turbulence or air refractive index gradients. Moreover, Liu et al. [26] used a high-speed rotating optical diffuser to eliminate the geometrical fluctuations of the laser beam. Those setups could all improve the measurement accuracy, but at the same time have the disadvantage that they are relatively complex and, besides the system with the parallel beam, do not compensate for any errors happening after the emission of the laser light.

Li et al. [27] used a digital optical phase conjugation system in a straightness measurement setup to compensate for the influence of beam bending due to an air refractive index gradient and turbulences in the measurement path, strongly decreasing the measurement deviation. As this setup is complex, using active components like a spatial light modulator, it is not suitable for a simple triangulation setup. Furthermore, a device would be necessary on the measurement surface to monitor the laser beam’s wavefront. Dobosz et al. [28] measure the temperature along the beam in an interferometric distance measurement system to account for variations in the refractive index.

To avoid the influence of speckle noise due to rough surfaces, a moving laser was introduced by Zbontar et al. [29], projecting a symmetrical laser spot pattern on the measurement surface. As this setup uses moving parts it is rather unstable and not very robust. Faulhaber et al. [30] used a spatial light modulator to project several spots on the measurement surface for the reduction of speckle noise, which is, however, complex and costly.

The effects of thermal expansion of a camera on measurements have been proven and quantified [31,32,33], and Pedersen et al. [18] calculated the influence of thermal expansions but did not compensate them. Li et al. [34] used a multiple linear regression model to compensate for temperature effects, whereas Flores-Fuentes et al. [35] used a support vector machine for error compensation. In general, it is possible to avoid such errors by the implementation of an extremely robust setup using components with a low temperature expansion coefficient, such as Invar, thus leading to higher costs and a heavier setup.

The error introduced due to the laser spot center calculation can be decreased by improved image processing algorithms and by averaging over time or several laser spot positions. The latter can be done through the projection of several laser spot images onto the detector by using multiple lasers in a setup to increase the achievable sensor accuracy and avoid occlusions [36,37,38]. Moreover, a diffraction grating was used in front of the detector to generate multiple points [39,40]. Takushima et al. [17] used a micro-lens array to achieve a similar effect.

The problem of the previously proposed methods is that they often only are able to compensate for a specific part of the possible error sources. In this contribution, a new compensation method for a stabilized single point laser triangulation system for medium distances is designed, tested, and verified. The basic principals of laser triangulation and an error model are introduced in Section 2, highlighting the influence of small parameter changes onto the overall accuracy of an LTD. The proposed idea to compensate for possible errors is presented in Section 3, where simulations indicate the practicability of the design. The experimental setup and the obtained measurement results are shown and discussed in the subsequent section, proving the effectiveness of this compensation method. Thus, several errors are purposely induced to show the improvement of the new setup in static and dynamic measurements. Finally, the paper is concluded with a brief outlook on possible future research work in Section 5.

## 2. Fundamentals

Before showing our approach in detail the fundamental background of an LTD as well as an error model are introduced.

### 2.1. Laser Triangulation

**Figure 1:**
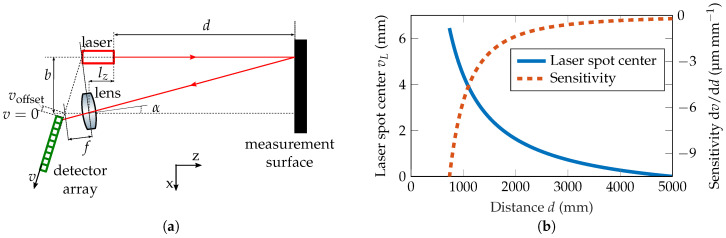
(**a**) Schematic of a laser triangulation setup consisting of a laser, a lens and a detector array in Scheimpflug condition. (**b**) Relation between the laser spot center $v_{L}$ and the distance *d* according to Equation (1). Sensitivity of the setup, defined by the shift of the laser spot center over the corresponding distance change. The detector is shifted in *v*-direction such that a maximum measurement distance of approximately 5000 mm is achieved ($v_{offset}\approx 1\;mm$).

Absolute and relative distances can be measured in a laser triangulation system using geometric relations. To do so, as can be seen in Figure 1a, a laser beam is emitted towards a diffusely reflecting measurement surface. A part of the scattered light is then imaged by a lens onto a 2D detector array, usually a charge-coupled device (CCD) or a complementary metal-oxide-semiconductor (CMOS) detector [14]. Hereby, the unknown distance *d* can be calculated according to

(1) $$d=\frac{f}{\cos\alpha }\;\cdot \;\left(1+\frac{\sqrt{b^{2}+{\left(b\;\cdot \;\tan\alpha -\frac{f}{\cos\alpha }\right)}^{2}}}{v_{L}+v_{offset}}\right)-b\;\cdot \;\tan\alpha +l_{z}.$$

The base distance *b* (distance between laser and lens center in *x*-direction), the lens offset $l_{z}$ (distance between laser and lens center in *z*-direction), the focal length *f* of the lens, the detector offset $v_{offset}$ (shift of the detector edge in *v*-direction), and the triangulation angle α (tilt of the lens) are known design parameters of the optomechanical setup, whereas the laser spot center $v_{L}$ on the detector is measured using image processing algorithms. To improve the accuracy of the setup, the lens and detector are placed under the so-called Scheimpflug condition [41], so that the plane of the detector and lens intersect at the same point with the laser line. This way, the laser spot is imaged as sharply as possible within the whole measurement range, which is defined by the field of view of the optical system. If the detector is moved in positive *v*-direction by $v_{offset}$, the minimal measurement distance increases, whereas the maximum measurement distance decreases.

The relation between the laser spot center position $v_{L}$ and the distance *d* can be seen in Figure 1b. This can be achieved either by a calculation with the known parameters according to Equation (1) or by calibration using measured data points. As the parameters are not exactly known due to production process tolerances and optical aberrations in a real optical system, typically a calibration process is necessary [42]. In the measurements in Section 4.2.2 a third order polynomial is used to calibrate the setup.

In this contribution, a measurement setup with a base distance of 130 mm, a lens with a focal length of 40 mm, and a detector with a pixel pitch of 1.67 μm is used to achieve the desired measurement range of 800–5000 mm. This leads to a sensitivity of around 3.2 px mm^−1^ at a measurement distance of 1 m, meaning that the laser spot center $v_{L}$ moves by 3.2 px on the detector if the distance *d* is changed by 1 mm. The distance is calculated by the movement of the laser spot center on the detector. Consequently, the calculation of this value determines and limits the performance of the overall measurement system [43]. It has been shown that centroid-based algorithms have several advantages, including fast calculation, good robustness, and high accuracy to a sub-pixel level [44]. In this work, a squared centroid algorithm with model-based circular segmentation is used [45].

### 2.2. Error Model in a Laser Triangulation System

In reality, the position of the laser spot center does not only move with a change in the distance but also due to different error sources. Due to thermal drifts, mechanical instabilities, and vibrations, the direction of the laser beam can vary significantly [46], which is also referred to as laser dithering. Consequently, the laser spot moves on the measurement surface and the detector, leading to a deviation in the measured distance [15].

The laser light travels twice through the air between the measurement device and the measurement surface. In the case of air turbulences or a temperature gradient, the laser beam may be deflected, leading to an error in the calculation of the distance [5]. This can be divided into jitter and beam bending, which is the dominating error source in long-distance measurements [27].

The coherent nature of laser light in combination with the roughness of the measurement surface leads to so-called speckles in the laser spot image, resulting in a measurement error [16,47,48]. Additionally, the wide range of possible surfaces with different optical properties [17,49,50] as well as the probes position and orientation towards the measurement surface limit the accuracy of LTDs [51].

The diffusely reflected light is eventually imaged by a lens and detector. If the lens or detector shift due to temperature changes or mechanical instabilities, the laser spot moves on the detector [18], leading to an error in the measured distance. The same would apply for deviations in the optical path calibration. Ma et al. [33] showed the image translation due to self-heating of the camera to be in the order of up to two pixels in experiments.

Last but not least, there is an additional error introduced in the calculation of the center position of the laser spot. This is caused by speckles, spatial quantization, analog-to-digital quantization as well as noise on the detector, e.g., photon shot noise or dark current noise [19,52]. As the laser spot center needs to be determined with sub-pixel accuracy to achieve the desired measurement precision, this also limits the overall accuracy of an LTD.

Additionally, it has been demonstrated that distance sensors exhibit a general drift over time due to temperature fluctuations [34,53,54]. Table 1 shows an exemplary overview and impact of several parameters in a laser triangulation setup with the previously given dimensions. Hereby, the introduced distance error Δd divided by the parameter change is denoted. The calculations are done using the paraxial approximation, assuming a perfectly thin lens without any aberrations and an infinitely small laser beam. The simulations are done using Zemax OpticStudio, taking into account the aberrations of the optical system as well as the extent of the laser beam. Consequently, there are slight differences between the values. As can be seen in Table 1, several parameters have a substantial impact on the measurement accuracy. Especially small changes in the laser angle as well as the detector and lens position can lead to significant deviations. As a temperature gradient cannot be simulated in the applied software, no value is given here. As the deviation with respect to the lens angle Δα is a result of the aberrations of a real lens, they do not have an influence on the calculations using the paraxial approximation. Thus, no value is given here.

Deviations of the laser angle of Δγ = 0.2°, respectively 3.5 mrad, as shown in Ref. [15], would result in a measurement deviation Δd of around 27 mm. A translation of the detector by 2 px in Ref. [33] is equivalent to a shift of 14.8 μm (pixel pitch of 7.4 μm). Consequently, this would lead to a measurement deviation of around 2.9 mm. With the base distance of 130 mm and the assumption, that the components are mounted on an aluminum base (coefficient of thermal expansion of 24 µm K^−1^ m^−1^), a temperature change of 1 ${}^{\circ }C$ would result in an increase of the base distance by 3 μm, leading to an error in the distance measurement of around 25 μm. Those calculations show that even small changes in the temperature or setup, in general, will lead to significant measurement errors. In order to increase the accuracy of a laser triangulation setup, these errors have to be avoided or compensated.

## 3. Optical Setup for Compensation of Measurement Errors

**Figure 2:**
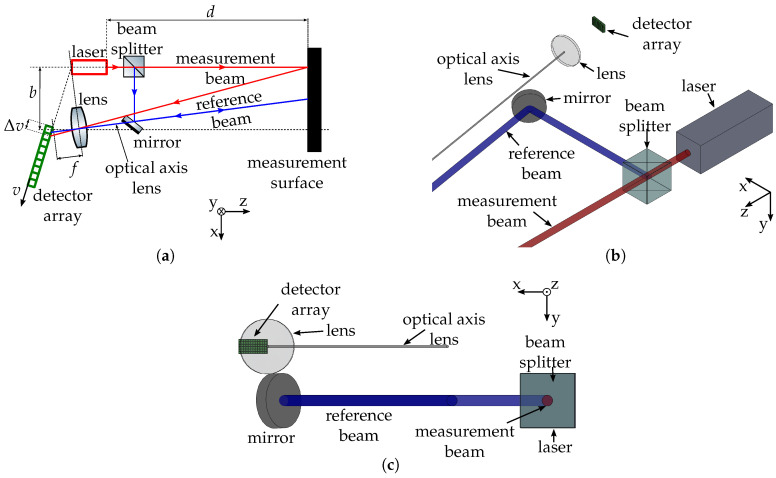
Schematic of a setup able to compensate for errors due to laser beam dithering, air refractive gradients and optomechanical thermal effects. (**a**) Schematic top view. (**b**) Isometric CAD view. (**c**) Frontal CAD view.

Several possibilities to compensate for errors have been listed in the introduction. As the previously proposed methods are either very complex or not capable of avoiding several errors simultaneously, a new compensation method is introduced here. The schematic setup can be seen in Figure 2, consisting of only an additional beam splitter and mirror. The laser beam is split up into a measurement and a reference beam, respectively. Those result in a measurement and a reference spot in the detector image, leading to the possibility to compensate for errors due to laser dithering, environmental conditions, and optomechanical shifts. Thereby, it is important that the reference beam is parallel to the optical axis of the lens. In this way, with a change of the distance *d*, the measurement spot center (MSC) $v_{meas}$ is changing, whereas the reference spot center (RSC) $v_{ref}$ is constant. In contrast, if $v_{meas}$ is altered due to an error, $v_{ref}$ changes in a similar fashion. Consequently, the deviation can be compensated by subtracting $v_{ref}$ from $v_{meas}$. The setup can either be constructed by placing the lens and detector above or below the laser in *y*-direction, as shown in Figure 2c. Alternatively, the beam splitter could also be tilted around the *z*-axis instead. Additionally, a penta prism beam splitter can be used to ensure that the reference beam is split at an angle of 90°.

**Figure 3:**
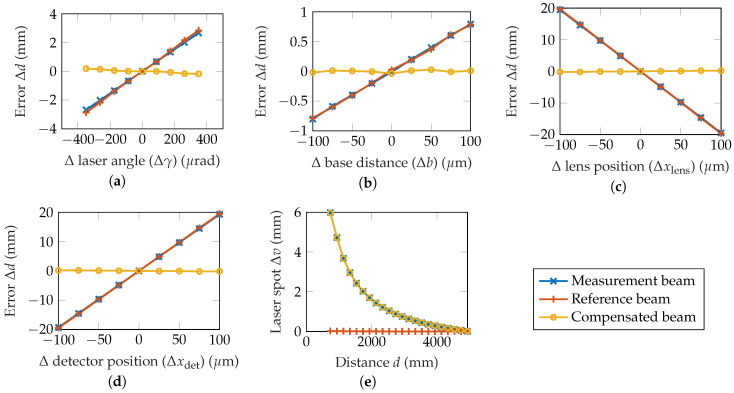
Simulated distance errors Δd of the measurement beam, the reference beam as well as the compensated beam ($\Delta d_{meas}-\Delta d_{ref}$) in dependence of a deviation of (**a**) the laser beam angle, (**b**) base distance Δb, (**c**) lens position Δx, (**d**) detector position Δx, and (**e**) measurement distance *d*.

The induced error Δd of the two beams as well as of the compensated beam is given in dependence of several parameters in Figure 3a–e. The error of the compensated beam $\Delta d_{comp}$ is calculated by $\Delta d_{comp}=\Delta d_{meas}-k\;\cdot \;\Delta d_{ref}$, with k=1 being the compensation factor. The results are obtained using a ray tracing simulation software. Therefor, the geometrical laser triangulation setup with its optical components is implemented in Zemax OpticStudio, and the detector image is obtained as the simulation output. Every geometrical parameter of the setup can then be varied iteratively to simulate the parameter changes reflected by the *x*-axis in Figure 3, e.g., the position of the lens or detector. This leads to a shift of the laser spot center in the simulated detector image, which can be transformed into a distance error Δd using the sensitivity of the setup. This approach has the advantage that optical aberrations and the extend of the laser beam are considered here.

The errors introduced in the previous section are compensated to a good degree (Figure 3a–d), whereas a change of the measurement distance is not affected (Figure 3e).

The errors with and without compensation are summarized in Table 2. The error is reduced by up to 99%. For the laser angle and the base distance, the achieved improvement is slightly lower. As can be seen in Figure 3a,b, this is due to the different displacement of the MSC and RSC because of the different paths the beams take. Consequently, depending on what parameter needs to be compensated, the compensation factor *k* has to be chosen accordingly. In a real measurement setup, it is not previously known what parameters are affected by drift, so *k* needs to be determined experimentally. It is important that the laser output, the beam splitter and the mirror stay in a fixed relation to one another, as a change in their geometrical relation would result in a deviation of only the reference beam, leading to an error in the compensated distance measurement.

This setup has several advantages to a simple laser triangulation setup or the previously mentioned setups. First of all, it only uses two standard optical parts and does not rely on active or movable parts. Secondly, this setup is able to compensate for errors due to laser beam dithering, changes in the air refractive index as well as thermal expansions in the optomechanical setup. Thus, a less stable and more cost-effective mechanical setup, laser, and detector might be used as the drifts of those components are eliminated. Yet the setup has one disadvantage. As the two beams are not emitted in parallel, the two points typically hit the measurement surface with some distance in between them. This does not affect the measured distance, as the reference beam is parallel to the optical axis of the receiver lens, but the measurement surface needs to be wide enough so that the reference beam is diffusely reflected as well and can consequently be detected by the sensor.

## 4. Experimental Results

The introduced setup has then been implemented in the laboratory to verify the simulations. After a brief description of the setup, the resulting measurements are examined.

### 4.1. Measurement Setup

The previously introduced setup was then implemented. The fiber-coupled laser diode has a center wavelength of 660 nm and a power of 10 mW (LasersCom, LDI-660-FP-10). It is focused by an achromatic lens with a focal length of 25 mm (Throl optics GmbH). This results in a laser spot diameter of about 3.5 mm at a distance of 1 m. To achieve the desired measurement range of 800–5000 mm, the laser spot is imaged using a biconvex lens with a focal length f=40 mm and a diameter of 31.5 mm (Linos AG), with an aperture diameter of 10 mm. The base distance *b* is set to 130 mm and the CMOS detector has a resolution of 3856 px×2764 px, with a pixel pitch of 1.67 μm (Dahua, DH-MV-A3A04MG10E). The viewing angle of the lens is set to 8°, whilst the detector is aligned according to the Scheimpflug principle. The laser is split up with a penta prism beam splitter with a relation of 50:50 (Artifex Engineering GmbH), whilst the reference beam is deflected by a Zerodur broadband dielectric mirror (Thorlabs, B111-E02). The beam splitter is tilted around the *z*-axis by 4° to spatially separate the two laser spots on the measurement surface.

With this setup, the desired measurement range is achieved. The theoretic relation between the laser spot center and the measurement distance can be seen in Figure 1b, as introduced before. The sensitivity of the setup is 5.1 px mm^−1^ at a distance of 0.8 m, 3.2 px mm^−1^ at a distance of 1 m, and 0.13 px mm^−1^ at a distance of 5 m.

### 4.2. Characterization of the Measurement Setup

The performance of this measurement setup is now characterized by different measurements. First of all, static measurements with a constant measurement distance *d* are conducted to investigate the temporal behavior of the setup due to thermal drifts. Afterward, errors are provoked to capture the efficiency of the compensation in static measurements. Finally, the measurement uncertainty in dynamic measurements over a measurement range of 910–1060 mm is determined.

#### 4.2.1. Temporal Behavior in Static Measurements

**Figure 4:**
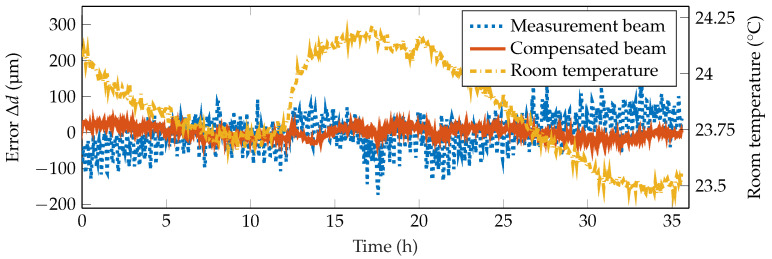
Measurement showing the compensation of drift effects. The measurement beam shows a stronger deviation than the compensated beam. A slight correlation of the measurement beam with the room temperature is visible.

First of all, this measurement setup is characterized in static measurements at a distance of 1 m. Thereby, the distance *d* is kept constant during the whole measurement. Theoretically, the laser spot center position should not move during the time. Due to the aforementioned drift of different parts, this is not true, as can be seen in a measurement of 36 h in Figure 4. Within this period, the measurement spot center changed by up to 1.12 px peak-to-peak. With the given sensitivity of 3.2 px mm, this equals a peak-to-peak distance error $\Delta d_{meas}$ of 350 μm. The correlation coefficient of the two spot centers yields a value of 0.96, meaning that they have a strong correlation. This can also be seen in the compensated laser spot center position, which only changes within a range of 0.28 px, resulting in a distance deviation of $\Delta d_{comp}=88\;\mu m$. Consequently, the static error of the long-term measurement is reduced by 75%. The drift of the signals can be explained by a slight change in the ambient temperature over time, as is indicated in Figure 4. As the correlation between the measurement laser spot center and the temperature yields a correlation coefficient of only −0.44, the introduced error could not be compensated to such a good degree using only the temperature information.

**Figure 5:**
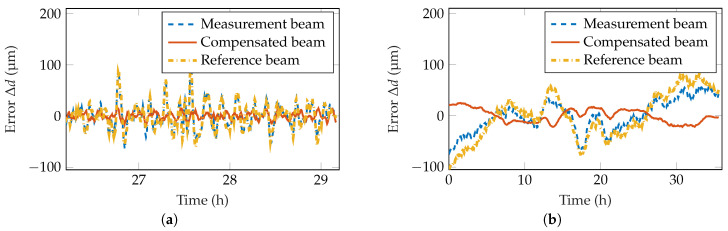
(**a**) Highpass-filtered data showing the compensation of short-term drift effects. A minimum-order filter with a passband frequency of 1.11 mHz, steepness of 0.85 and stopband attenuation of 60 dB was applied. (**b**) Lowpass-filtered data showing the compensation of long-term drift effects. A moving average filter with a window length of 1 h was applied.

To emphasize the compensation of both short-term and long-term drifts, Figure 5a,b show an excerpt of the highpass- and lowpass-filtered signals, respectively. For frequencies below a period duration of 15 min, it is clearly visible that the short-term variations of the signal have a high correlation of 0.96. Therefore, short term disturbances, e.g., due to laser beam dithering, can easily be compensated. Long-term drifts, which mainly happen due to temperature changes of the setup, have a similar correlation of 0.97, as can be seen in Figure 5b. Consequently, they can be compensated to a similar degree.

**Figure 6:**
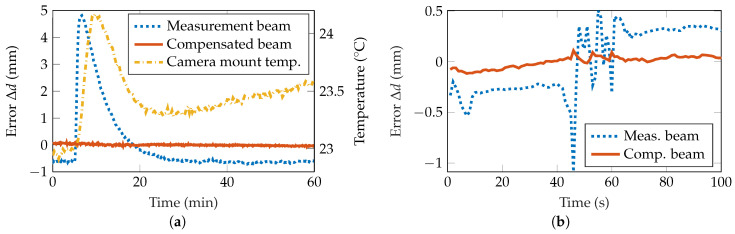
(**a**) A heating resistor at the camera mount was turned on after 6 min for a duration of 30 s. This results in a shift of the MSC due to the thermal expansion, whereas the compensated beam position is not affected. (**b**) Due to mechanical vibrations at the optical table the measurement beam position shows a movement, whilst the impact is strongly reduced in the compensated beam position.

Moreover, the thermal expansion of single components was examined. A heating resistor with a value of 330 Ω was attached to the mount of the detector. To monitor the temperature change, a temperature sensor was mounted next to it. The influence of the heating up can be seen in Figure 6a. When a power of 1.7 W is dissipated in the resistor at t=6min for 30 s, the surrounding material is heated up by around 1 °C. As a consequence, both the measurement beam and the reference beam deviated by around 17.72 px, leading to a distance change $\Delta d_{meas}$ of about 5.5 mm whereas the deviation of the compensated beam is reduced to 0.48 px or 0.15 mm. Thus, the introduced error is decreased by 97%. As this knowingly only affects the position of the detector, the compensation factor was set to 1, as has been proven as the ideal compensation factor for a sole movement of the detector in the simulations. If a compensation factor of 0.8 is applied, as in the other experiments, the deviation of the compensated beam is still reduced to $\Delta d_{comp}=1.06\;mm$, resulting in an improvement of 78%.

In additional experiments, vibrations of the measurement setup were provoked to test the ability of the setup to compensate for errors. The optical table on which the measurement setup is placed was mechanically moved. The resulting displacement of the measurement beam position and the compensated beam position is shown in Figure 6b. Whereas the measurement beam deviates by up to 5.1 px or 1.59 mm peak-to-peak, the compensated beam deviation is reduced to 0.7 px or 0.22 mm. Consequently, the deviation is reduced by 86%.

#### 4.2.2. Dynamic Measurements

**Figure 7:**
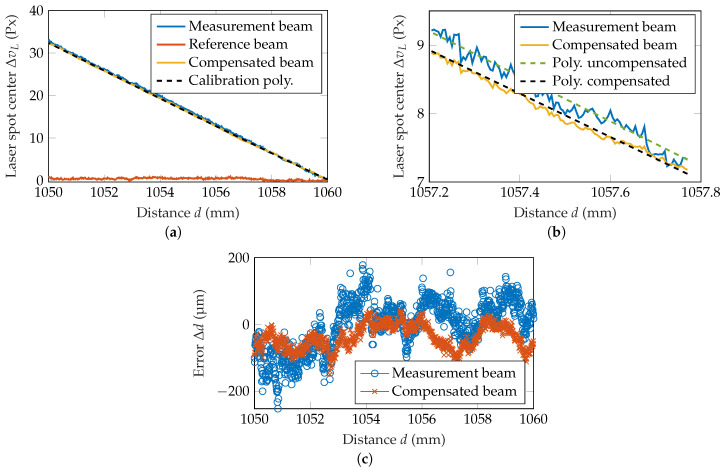
(**a**) Dynamic measurement over the range of 10 mm. (**b**) Close-up on part of the measurement, highlighting the improvement using the compensated beam. (**c**) Measurement deviation of the compensated and uncompensated beam.

In subsequent measurements, the improvement of the compensation setup in a distance measurement is shown. The measurement surface is moved in the range of 1050–1060 mm with a step size of 5 μm, driven by a linear translation stage with an on-axis accuracy of 5 μm (Thorlabs, NRT150). Thereby, the position of the measurement beam is dependent on the measurement distance *d*, while the position of the reference beam stays relatively constant, as can be seen in Figure 7a. Consequently, the compensated beam shows the same behavior as previously introduced in Figure 3e, opening up the possibility to measure the absolute distance. A calibration polynomial of third order is calculated for both the compensated and uncompensated beam, which can be used to transfer the measured and compensated laser spot center positions into distances.

In Figure 7b, it already is visible that the uncompensated beam dithers stronger around the calibration polynomial. In a period of three days, 18 dynamic measurements were recorded. The deviation of the distance measurement Δd can then be calculated by subtraction of the true distance $d_{true}$ from the measured or compensated distance $d_{meas}$ or $d_{comp}$. A comparison of the deviation between measurements using the measurement beam and the compensated beam is given in Figure 7c. As it is clearly visible, the deviation of the measurement beam is bigger compared to the deviation of the compensated beam. As a single measurement takes around 3 h, a drift of the error can be explained with a drift of the laser spot center as shown in the static measurements before. An error of 200 μm roughly equals to a laser spot center shift by 0.6 px, which was the typical drift amplitude in the previous measurements (see Figure 4). This drift can be compensated to a good degree. Using this deviation, a root mean square error (RMSE) can be calculated, characterizing the performance of the measurement setup. Within the 18 measurements, the average RMSE is reduced from 77 μm with the uncompensated beam to 61 μm with the compensated beam, which equals a reduction of 22%. This improvement is not as good as it has been proven in simulations and static measurements because speckles are another major error source in laser triangulation measurements. However, the compensation setup still leads to a significant improvement in the RMSE of the distance measurement.

**Figure 8:**
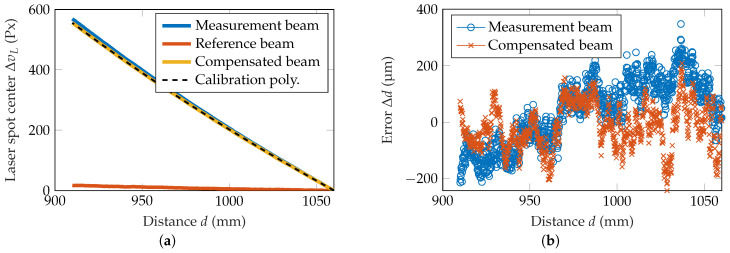
Dynamic measurement in a range of 150 mm. (**a**) Comparison of the measurement, reference and compensated beam position. (**b**) Measurement deviation for the measurement and compensated beam.

In the last experiment, 15 dynamic measurements in a range of 910–1060 mm with a step size of 50 μm were conducted within five days, which equals the maximum possible translation of the stage. The resulting laser spot movement for one measurement can be seen in Figure 8a. As the reference beam is not perfectly aligned with the optical axis of the lens, the reference beam laser spot center slightly moves with the change of the distance. Consequently, the compensated beam also shows a slight deviation to the measurement beam. Within the 15 measurements, the average RMSE is reduced from 109 μm with the measurement beam to 85 μm with the compensated beam, which equals a reduction of 22%. The improvement is the same as it has been for the translation within 10 mm before. Yet again, the deviation of the compensated beam clearly decreased, as can be seen in Figure 8b.

## 5. Conclusions

In this contribution, a compensation setup for a laser triangulation device was introduced. The laser beam is split up with a simple optical setup consisting only of a beam splitter and mirror. The reference beam is directed in parallel to the optical axis of the imaging lens, resulting in a reference spot center position independent of the measurement distance. Thereby, errors resulting from laser dithering, beam bending, and optomechanical deformations can be compensated. This was shown through simulative investigations as well as measurements. As a consequence, the deviation in static measurements was reduced by 75%, while the measurement uncertainty in dynamic measurements was reduced to 85 μm at a distance of 1 m.

A current problem of the setup is that mechanical shifts of the beam splitter or the mirror lead to an additional error that can not be compensated. As a result, those two components have to be fixed more stable, e.g., by using special temperature stabilized optical mounts made from stainless steel. With this further stabilized setup, an extensive characterization of the laser triangulation device over its complete measurement range is in the scope of subsequent investigations. During such experiments, an ideal compensation factor has to be determined. Moreover, the effects of the compensation of errors due to different surface materials, a measurement surface tilt, varying laser intensity, different types of lasers, and other error sources need to be examined, as the compensation setup could also be potentially effective for them.

## Figures and Tables

**Table 1:** Relationship between distance error Δd and parameter changes at a measurement distance of d=1 m. For example with a change of the laser angle by Δγ=1 μrad, the calculated distance changes by Δd=7.7 μm.

Category	Parameter	Calculation	Simulation
**Laser dithering**	Laser angle Δγ	7.7 μm/μrad	7.6 μm/μrad
**Beam bending**	Temperature gradient	3.6 μm/(K/m)	- - -
**Optomechanical Setup**	Base distance Δb	7.8 μm/μm	8.0 μm/μm
	Lens position $\Delta x_{lens}$	195 μm/μm	195 μm/μm
	Lens position $\Delta z_{lens}$	25.8 μm/μm	23.4 μm/μm
	Lens angle Δα	- - -	0.7 μm/μrad
	Detector position $\Delta x_{det}$	193 μm/μm	193 μm/μm
	Detector position $\Delta z_{det}$	24.10 μm/μm	22.3 μm/μm

**Table 2:** Relationship between distance error Δd in dependence of parameter changes at a measurement distance of d=1 m.

Parameter	Uncompensated	Compensated	Reduction by
Δ laser angle (Δγ)	7.6 μm/μrad	0.53 μm/μrad	93.1%
Δ base distance (Δb)	8 μm/μm	0.32 μm/μrad	96.0%
Δ lens position ($\Delta x_{lens}$)	195 μm/μm	2 μm/μm	99.0%
Δ detector position ($\Delta x_{det}$)	193 μm/μm	2 μm/μm	99.0%
